# Exploring the Efficacy of Endoscopic Ventriculostomy for Hydrocephalus Treatment via a Multicompartmental Poroelastic Model of CSF Transport: A Computational Perspective

**DOI:** 10.1371/journal.pone.0084577

**Published:** 2013-12-31

**Authors:** John C. Vardakis, Brett J. Tully, Yiannis Ventikos

**Affiliations:** 1 Institute of Biomedical Engineering and Department of Engineering Science, University of Oxford, Oxford, United Kingdom; 2 Oxyntix Ltd., Department of Engineering Science, University of Oxford, Oxford, United Kingdom; 3 Department of Mechanical Engineering, University College London, Torrington Place, London, United Kingdom; Tokyo Metropolitan Institute of Medical Science, Japan

## Abstract

This study proposes the implementation of a Multiple-Network Poroelastic Theory (MPET) model coupled with finite-volume computational fluid dynamics for the purpose of studying, in detail, the effects of obstructing CSF transport within an anatomically accurate cerebral environment. The MPET representation allows the investigation of fluid transport between CSF, brain parenchyma and cerebral blood, in an integral and comprehensive manner. A key novelty in the model is the amalgamation of anatomically accurate choroid plexuses with their feeding arteries and a simple relationship relaxing the constraint of a unique permeability for the CSF compartment. This was done in order to account for the Aquaporin-4-mediated swelling characteristics. The aim of this varying permeability compartment was to bring to light a feedback mechanism that could counteract the effects of ventricular dilation and subsequent elevations of CSF pressure through the efflux of excess CSF into the blood system. This model is used to demonstrate the impact of aqueductal stenosis and fourth ventricle outlet obstruction (FVOO). The implications of treating such a clinical condition with the aid of endoscopic third (ETV) and endoscopic fourth (EFV) ventriculostomy are considered. We observed peak CSF velocities in the aqueduct of the order of 15.6 cm/s in the healthy case, 45.4 cm/s and 72.8 cm/s for the mild and severe cases respectively. The application of ETV reduced the aqueductal velocity to levels around 16–17 cm/s. Ventricular displacement, CSF pressure, wall shear stress (WSS) and pressure difference between lateral and fourth ventricles (ΔP) increased with applied stenosis, and subsequently dropped to nominal levels with the application of ETV. The greatest reversal of the effects of atresia come by opting for ETV rather than the more complicated procedure of EFV.

## Introduction

Hydrocephalus (HCP) can be succinctly described as the abnormal accumulation (imbalance between production and circulation) of CSF within the brain [1]. This balance of CSF production and reabsorption allows the maintenance of the CSF pressure to lie within the approximate range of 600–2000 Pa [2]. HCP cannot be considered a singular pathological entity, but instead, a consequence of a variety of congenital and acquired disorders present within the CNS [3]. HCP is classified with regards to whether the point of CSF obstruction or discreet lesion lies within the ventricular system (obstructive) and obstructs the flow before it enters the subarachnoid space [4], or not (communicating). It has no known cure and current treatment methods display an unacceptably high failure rate. The two prominent treatment methods – shunt implants and endoscopic third ventriculostomy – are both surgical interventions that are statistically indistinguishable in terms of available clinical survival data and efficacy of short or long term success [5], [6]. Ventricular shunting suffers from various complications [3], [7], high failure rates (up to 50% after two years) and high average costs [7]–[9]. There are therefore strong incentives for neurosurgeons to find alternative modes of intervention.

### CSF Production, Transport and Absorption

**Figure 1:**
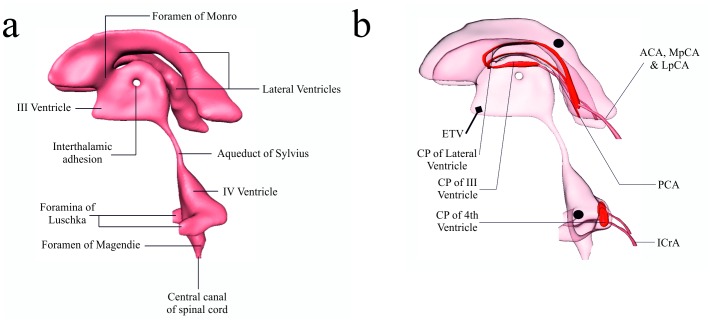
The cerebroventricular system, along with the choroid plexuses and some feeding arteries. (a) CSF circulates through the four brain ventricles and in the subarachnoid space surrounding the brain and spinal cord. (b) View of the ventricular system along with the choroid plexus (CP) of the lateral (LV), 3^rd^ and 4^th^ ventricle. The arteries supplying these plexuses (not all are presented in the figure) are the anterior and lateral posterior choroidal arteries (ACA & LpCA), the P2 segment of the posterior cerebellar artery (PCA) and the medial posterior choroidal artery (MpCA) [12]. The MpCA arises from the PCA (or its branches) and enters the roof of the third ventricle [74] which feeds the CP situated there. Finally, the CP of the fourth ventricle is supplied by the posterior inferior cerebellar artery (PICA), inferior cerebellar artery (ICA) and superior cerebellar artery (SCA) [75]. ETV is the location of perforation of the floor of the third ventricle during endoscopic third ventriculostomy. [•] represents the locations of measurement for the deduction of the pressure difference between the lateral and fourth ventricles.

Figure 1a illustrates the ventricular cavity under consideration in this work. In adults, the CSF volume is estimated to be approximately 150 *ml*, where only 25 *ml* are segregated to the ventricles and the remainder occupies the cranial and subarachnoid spaces [10]. When considering adults, the normal CSF circulation proceeds in a consistent and uniform manner since this flow regulation is primarily responsible for cerebral homeostasis [10], [11]. The classical hypothesis of CSF formulation involves the production of CSF at the choroid plexus (CP) of the lateral, third and fourth ventricles. The CP’s possess the added impediment of being the most complicated vascular structures in the brain [12]. The choroid plexuses of the lateral ventricles are fed from various arteries. Some are described in Figure 1b. Exact production proportions are still in the speculative phase, as it is still unclear as to how extensive both choroidal and extrachoroidal CSF production is [13]–[16].

In this work we also consider the effects of including aquaporin-4 (AQP4) within our mathematical framework. It is the most predominant aquaporin in the brain, and is located on the external and internal glial limiting membranes, the basolateral membrane of ependymal cells [17] and astrocytes. In the latter, AQP4 occupies three key locations, namely the perivascular astrocyte end feet, perisynaptic astrocyte processes and in processes that involve K^+^ clearance, such as nonmyelinated axons and the nodes of Ranvier [18]. This tactical distribution suggests that AQP4 controls water fluxes into and out of the brain parenchyma. AQP4 has been deemed to possess the essential role of controlling the water balance in the brain [19].

CSF is secreted continuously; however, findings indicate strong circadian variations to its production thought to be due to the finely regulated responsibilities of the autonomic nervous system [10]. Around 600 ml of CSF is produced each day, with a CSF volume turnover of four [10], [11], [20]. CSF travels from the sites of secretion to the sites of absorption via a unidirectional rostrocaudal flow regime within the cerebral ventricles. It begins to circulate in the cerebroventricular system by flowing out of the lateral ventricles via the foramen of Monro and into the third ventricle. From there the CSF passes to the fourth ventricle via the aqueduct of Sylvius whereby the foramina of Magendie (medial, single) and the bilateral foramina of Luschka act as the final outlets of the CSF leading to the subarachnoid space [21]. The central canal of the spinal cord also receives CSF from the fourth ventricle; however, in comparison to the foramina of Luschka and foramen of Magendie, this is a minute quantity. Once the CSF has exited from the cerebral ventricular system, it accumulates in the subarachnoid cisterns surrounding the brainstem before it gets reabsorbed [11]. Experiments suggesting the existence of extrachoroidal CSF production include proof that there is a reduction, but not elimination, of CSF when surgical choroid plexotomy takes place [22]. The capillary-astrocyte complex in the blood-brain barrier is understood to be an active producer of interstitial fluid (ISF) [23].

CSF absorption is more complex than the existing classical theory just outlined. As opposed to the highly orchestrated transport process of CSF secretion, CSF absorption occurs via other outflow mechanisms, such as along the sheaths of major blood vessels and cranial nerves [13]. New functionally intimate links between cerebral interstitial fluid, CSF and extra cranial lymph have been identified [24]. The glymphatic pathway possesses both a lymphatic like system and revolves around AQP4 dependent astroglial water flux which is responsible for the clearance of ISF, CSF and solutes from the parenchyma [25]. Neuroprotective fluctuations of the abundance of AQP4 in hydrocephalic rat brains which may contribute to stabilizing the ICP have also been recorded [26]. Studies have indicated that CSF absorption also occurs along the spinal nerves [27] and across capillary aquaporin channels [28], [29]. The ‘minor pathway’ present in small mammals and developing immature brains in humans has been recently outlined as a way of classifying HCP [30].

### Endoscopic Third and Fourth Ventriculostomy (ETV and EFV)

Endoscopic third ventriculostomy (ETV) was initially restricted to patients with triventricular HCP, where a bulging translucent floor of the third ventricle floor was evident and with a patient age threshold of greater than two years [31]. In recent years, the list of possible suitable candidates has increased dramatically, especially patients which exhibit a lack of communication between the ventricles and the subarachnoid space. In addition, the patient age requirement has been relaxed [32]. Many recommend that ETV be suggested as a first-line treatment to all patients that require management of HCP [31]–[36]. When compared to shunts, ETV also lacks problems in the domains of disconnection, occlusion, high infection rate, overdrainage and valve dysfunction [7], [32].

A special case of atresia of the foramina of the fourth ventricle was witnessed by Gianetti et al. [37], where prior shunting was unsuccessful and where the size of the foramen of Monro and third ventricle prohibited the planning of an ETV procedure [37]. A burr hole was created as in ETV and the fourth ventricle was approached via the cerebellar hemisphere. Translucent membranes were seen to occlude the aforementioned foramina (atresia). EFV has the advantage in that all three CSF exits in the fourth ventricle can be opened, irrespective of the level of ventricular dilation or contraction of the rest of the ventricular system. EFV may prove to be a sound alternative (since ETV is considered as the preferred option for various types of Fourth Ventricular Outlet Obstruction (FVOO) [38]) in cases where ETV is not available [33].

### Existing Models of the CSF Compartment

For relevant overviews on CSF hydrodynamics and their applications, the reader may refer to the work of Dutta-Roy et al. [39], Howden et al. [40], Kaczmarek et al. [41], Kurtcuoglu et al. [42], Levine [43], Linninger et al. [14], Smillie et al. [44] and Sivaloganathan et al. [45].

The aim of the paper is to justify a means by which derivative pathologies of HCP (ventricular enlargement due to aqueductal stenosis or FVOO) can be alleviated via two surgical means, namely endoscopic third and fourth ventriculostomy. A computational comparison of the two techniques is presented using a novel application of Multiple-Network Poroelastic Theory (MPET) to investigate cerebral fluid transport. A detailed investigation of multiscalar, spatio-temporal transport of fluid between the cerebral blood, cerebrospinal fluid (CSF) and brain parenchyma is conducted. Specifically, the MPET model of the cerebral tissue is coupled with a three-dimensional representation of the CSF flow patterns arising from the source of production (CP) within a patient-derived cerebroventricular system.

## Methods

### Anatomy Acquisition

**Figure 2:**
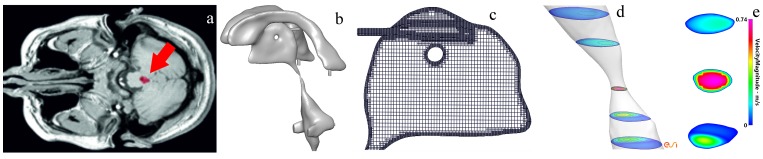
The process of preparing the anatomically accurate ventricular system, choroid plexuses and respective arteries. (a) Manual 3-D segmentation of the cerebral ventricles using Amira. The red arrow indicates a highlighted region representing part of the cerebral aqueduct. (b) The outcome of the smoothing operations undertaken by Blender on the STL file. The same software is also used to artificially apply the varying degrees of aqueductal stenosis. (c) CFD-VisCART is used to generate the grid. (d) CFD-VIEW is used as the post processing tool to analyze the results. (e) The velocity magnitude at different points on a severely stenosed aqueduct.

Imaging was performed on a 1.5T GE Signa system (Waukesha, WI, USA) and a T2 weighted imaging sequence was used for obtaining the brain anatomy data (Figure 2a). Images were acquired axially covering the whole brain at a voxel size of 1×0.5×2 mm^3^. These were then interpolated to 0.5 mm isotropic voxel size volume data which was used as an input for further processing.

The acquired voxels were manually segmented for the ventricular system using Amira (Mercury Computer Systems, San Diego, CA, USA) and the raw segmented geometry from this process was converted to a Stereo Lithography (STL) file. In order to preserve key anatomical features such as the Sylvian aqueduct, subsequent smoothing of the STL file was done using the open-source modelling software, Blender (The Blender Foundation, www.blender.org). The geometry can be seen in Figure 2b. Mesh generation (Figure 2c) and post processing (Figure 2d–e) are discussed in the *Solution Method and post processing* section.

### Multiple-Network Poroelastic Theory (MPET)

In geomechanics and more specifically fractured rock, an MPET medium can be considered as a solid matrix percolated by low porosity pores and high porosity fissures. These pores and fissures are in communication with each other via the transfer of fluid. MPET theory amalgamates porous flow laws, conservation of mass, Terzaghi effective stress and stress-strain relationships [1], [46]. Representing these in a convenient vector form:
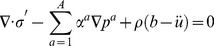
(1)

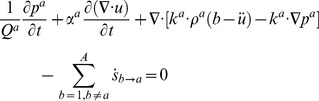
(2)


The forms of the simplified governing equations that will be used to expand to the quadruple MPET model used in this study are in the form of (1) and (2).

### Biological MPET Description

The MPET theory will revolve around the quadruple-MPET model. This means letting 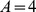 in equations 1 and 2. Biologically, this is derived by accounting for an MPET system that accommodates a high pressure arterial network (*a*), lower pressure arteriole/capillary network (*c*), extracellular/CSF network (*e*) and finally a venous network (*v*).

In the ensuing discussion, a spherical representation of the brain geometry will be utilised [44]. This allows the mechanical properties of concern to be preserved, whilst simultaneously simplifying the model. For this study, we continue to use the data selected by the current state-of-the-art as summarized in Table 1.

**Table 1:** Values used in the MPET model [41], [44], [52], [76]–[78].

Parameter	Value	Units	Parameter	Value	Units
***r_v_***	30×10^−3^	*m*	^⇑^ ***s_ac_***	1.5×10^−19^	*m^2^N^−1^s^−1^*
***r_M_***	100×10^−3^	*m*	^⇑^ ***s_cv_***	1.5×10^−19^	*m^2^N^−1^s^−1^*
***L***	70×10^−3^	*m*	^⇑^ ***s_ev_***	1.0×10^−13^	*m^2^N^−1^s^−1^*
***d***	3, 1.25,0.8 (×10^−3^)	*m*	^⇑^ ***s_ce_***	2.0×10^−19^	*m^2^N^−1^s^−1^*
***E***	584	*Nm^−2^*	***κ^a,c,v^***	1.0×10^−10^	*m^2^*
***p_bp_***	650	*Nm^−2^*	***κ^e^***	1.4×10^−14^	*m^2^*
***p_bpA_***	13.3×10^3^	*Nm^−2^*	***K***	668	*Nm^−2^*
***R***	8.5×10^13^	*m^−3^*	***λ***	524	*Nm^−2^*
***Q_p_***	5.8×10^−9^	*m^3^s^−1^*	***G***	216	*Nm^−2^*
***Q_o_***	5.8×10^−9^	*m^3^s^−1^*	***α^a,e,c,v^***	1.0	
***µ^e^***	8.9×10^−4^	*Nsm^−2^*	***ν***	0.35	
***µ^a,c,v^***	2.67×10^−3^	*Nsm^−2^*	***β***	0.99	

The parenchyma of an adult brain is represented as a spherical shell. The outer radius of this shell is given by 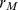, whilst the lumped representation of the lateral ventricles are represented by another spherical shell with radius 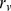. This is an adult brain that is being taken under consideration; hence a rigid wall approximation can be envisaged stemming from the elimination of layers like the dura mater and scalp. CSF is assumed to be produced at a constant rate (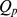) within the ventricles. The third and fourth ventricles along with the Sylvian aqueduct are assumed to be connected to the SAS. The parameters *μ^p^*, *κ^p^* and *α^p^*_,_ where *p = a,e,c,v* (described above) represent the viscosity, permeability and Biot parameters of the interstitial fluid networks. The parameters  denote network transfer coefficients, 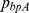 and 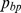 are the arterial blood pressure and blood pressure in the sagittal sinus respectively. The geometry representing all the features of the ventricles arise from a volunteer scan already outlined. The four-compartment MPET model is governed by the following system of equations, where *a* in equations 1 and 2 takes the form of 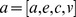.

Assuming a linear stress-strain relationship and isotropic permeability, the effective stress is represented by Hooke’s law, and is inverted for stress and subsequently represented as a function of displacement with the aid of Einstein notation to calculate divergence:
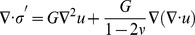
(3)


From the above, *G* is the shear modulus and is equivalent to 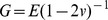, where *E* is the Young’s modulus and *v* is the Poisson ratio. An isotropic permeability would imply 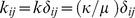. The final system of equations utilises a one-dimensional, spherically symmetric geometrical representation, which is provided by the transformation of the nabla (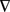) operator in spherical coordinates. In the context of the biological system under consideration, the following assumptions are also made to simplify the nabla transformation: 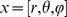, 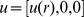
and 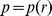. The aforementioned transformations for the nabla representation are used in place of 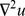,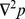, ∇⋅*u*, ∇(∇⋅*u*), ∇⋅*b* and 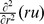.

Additional assumptions include the notion that HCP has a long time scale for development, of the order of days, weeks or even years (if one considers the chronic case). It is therefore evident that an assumption of a quasi-steady system is not unrealistic. It should be noted that the MPET model does not take pulsatile effects into account.

**Figure 3:**
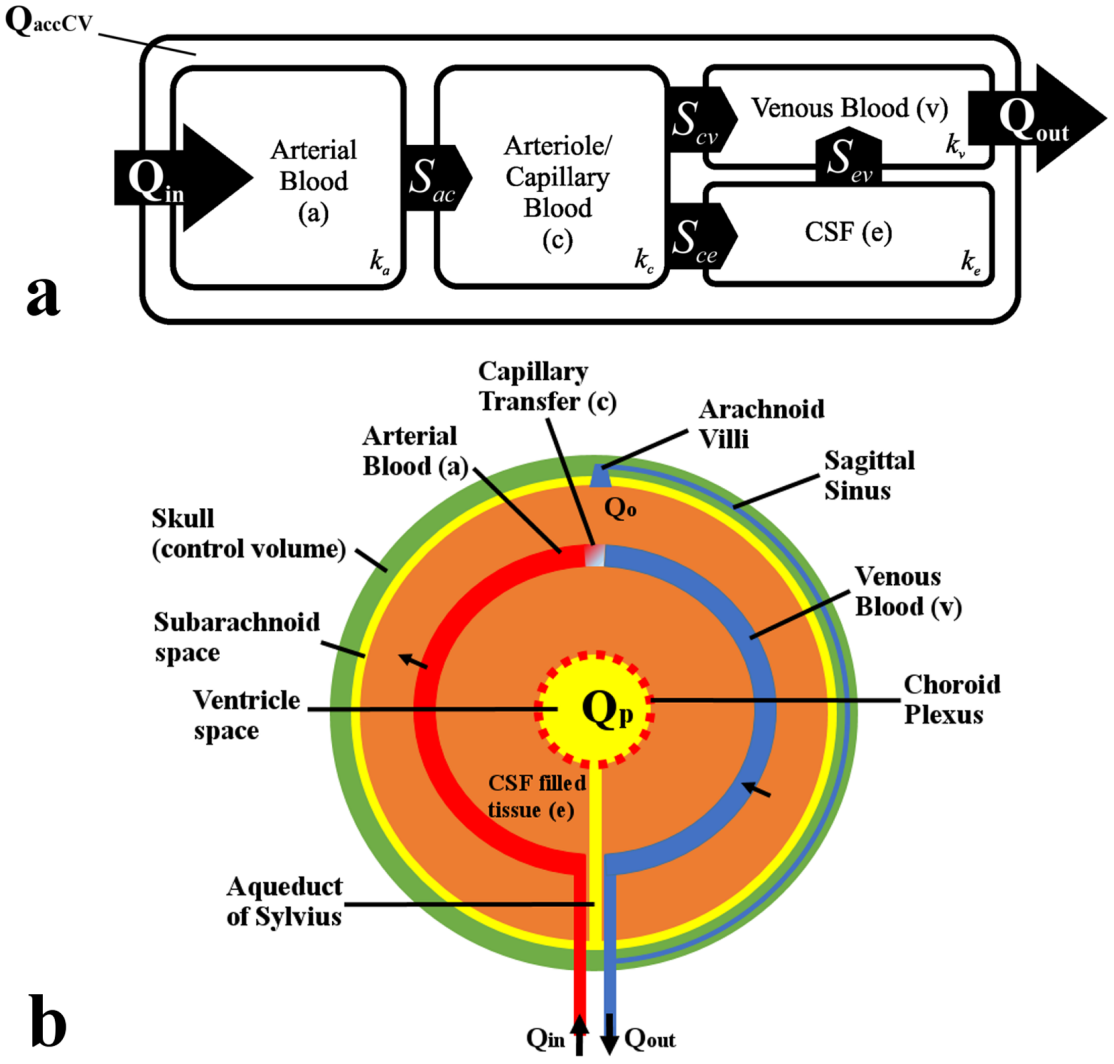
A schematic of the quadruple MPET model and its simplified spherical representation. (a) The above schematic represents the transfer restrictions placed on the MPET model. For example, it can be seen that flow is prohibited between the CSF and arterial network [1]. (b) The parenchyma of the brain is simplified to a spherical shell. In our MPET model, the ventricles are connected to the subarachnoid space via an anatomically accurate representation of the ventricular system. The two arrows in the figure denote directional transfer between arterial blood and CSF filled tissue (*S_ce_*), and finally CSF filled tissue and venous blood (*S_ev_*).

An insight into the specifics of continuity and inter-compartment transport for the system is also important. It is necessary to restrict the transfer of CSF between fluid networks, and Figure 3 sketches the setting in which the quadruple MPET model functions. From Figure 3a, it can be seen that:
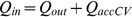
(4a)

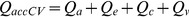
(4b)

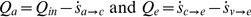
(4c)

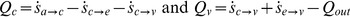
(4d)


From equations 4a–4d, it is noted that *Q_in_*, *Q_out_* and *Q_accCV_* represent the flux entering, leaving and accumulating in the control volume. The rest of the flux transfer terms have been explained in the earlier parts of this chapter, and are driven by a hydrostatic pressure gradient.

More segregated assessments on the transfer include: i) Transfer of flux occurs between the arterial and capillary network on an on-going basis, hence 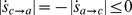 ii) Fluid flux from the CSF is transferred to the venous compartment, 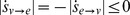 iii) Fluid transport from the capillary network will feed either the CSF or venous compartment, hence 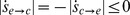 or 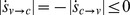 iv) No fluid transfer between arterial, CSF or venous compartments, 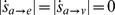. The convention is that 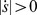 represents a loss of fluid from the network in question. The equations that will be solved in order to investigate HCP are displayed below, along with the new adjustments just described. We discuss boundary conditions and discretization specifics for these equations.
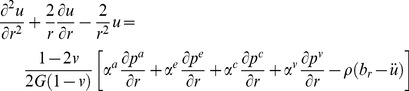
(5a)

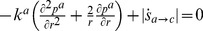
(5b)

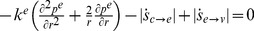
(5c)

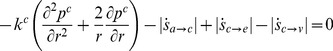
(5d)

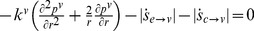
(5e)


In equations 5a–5e, the transfer between networks is via a hydrostatic pressure gradient, hence 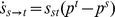. Here, 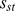 is a constant scaling the flow between networks *s* and *t*. The values of the Biot parameters, permeabilities and transfer coefficients are given in Table 1. Each of the ten constants was varied four times, through several orders of magnitude, in an initial parametric study of 4^10^ permutations. The details are outlined in Tully & Ventikos [1].

The following relationship was proposed to alleviate the constraint of a unique permeability for the CSF compartment in order to account for AQP4′s swelling characteristics:
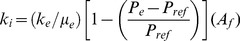
(6)


From equation 6, *k_e_* and *µ_e_* have already been described, *P_e_* is the CSF pressure, *P_ref_* is a reference pressure and finally *A_f_* is an amplification factor, and here it has a value of unity. The permeability was made to vary between the interval: 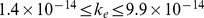, and coupled with the varying CSF pressure *P_e_*. *P_ref_* was chosen to possess a value *P_ref_* = 1 *kPa*. Due to insufficient experimental data, AQP4 expression is not accounted for in equation 6. More detail can be assigned to this boundary condition (when sufficient data becomes available) by including the swelling characteristics as a function of pressure and aquaporin expression. This can be done by additionally altering the relevant transfer coefficient 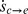 in equations 5c and 5d.

Finally, we define a useful parameter, namely the increment of fluid content, ζ, in terms of strain, ε as:
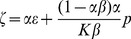
(7)


In equation 7, ε, the dilation, is given by 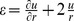, β is Skempton’s coefficient and *K*, the bulk modulus, is given by 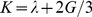. β essentially denotes the measure of the distribution of the applied stress between the solid matrix and CSF. A value of β = 0.99 represents a saturated mixture where the applied load is nearly entirely supported by the CSF fluid. The values of *K*, *λ* and *G* are given in Table 1.

### Boundary Conditions for the Skull

As discussed earlier, the shell responsible for outlining the skull lies at 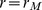, and here the displacement is deemed to be naught, as we are taking into account a rigid adult skull.
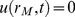
(8)


The blood pressures in the arterial and venous compartments are assigned physical values:
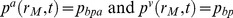
(9)


In addition to the above boundary conditions, there is a restraint on the flow both into and out of the capillary network (*c*) at the skull and the resulting accumulation of CSF into the venous network ultimately results in a pressure rise, hence:
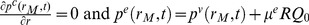
(10)


From equation 10, *µ_e_* is the CSF viscosity, *R* is the resistance due to arachnoid granulations and finally *Q_o_* is the efflux of CSF at the region of the skull.

### Boundary Conditions at the Ventricular Wall

Stress is assumed continuous across the wall, Hence at 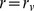,
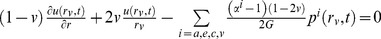
(11)


A pressure drop exists in the capillary compartment due to the production of CSF from the blood, hence:
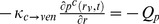
(12)


From equation 12, 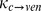 represents the capillary network resistance to the flow from the capillary network, and has a value of 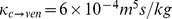 [1], [46].

There is no flow into or out of the arterial and venous networks, hence:
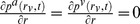
(13)


The final boundary condition involves the conservation of mass of fluid within the ventricular system (*d* is the diameter of the aqueduct), and more specifically, it represents the amount of CSF produced by the porous choroid plexuses:
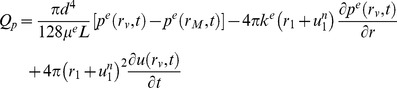
(14)


### Boundary Conditions at the Choroid Plexuses and Outlets

The porosity of the choroid plexus was varied according to the constant initial inlet pressure of 2000 Pa. An outlet pressure of 0 Pa was assigned to all exits from the fourth ventricle. In addition, this surface was assumed isotropic, with a permeability of 10^−10^ m^2^. The choroid plexuses shown in Figure 1 are supplied by the aforementioned unified initial inlet pressure boundary condition representing the feeding arteries at the different choroid plexus locations that ultimately produce CSF filtrate. The CSF produced in the plexuses passes through the choroid plexus surface (which encapsulate both the basolateral and apical membranes) of varying porosity and aforementioned permeability.

These conditions are assigned to the surface. In addition, the capillary network resistance given by *κ_c→ventricle_*, is included in the framework of the choroid plexuses producing CSF filtrate via the inlet blood supplies.

When running the CFD simulations to determine the CSF flow dynamics through the ventricular system, the flux of CSF exiting the outlets is used to replace the Poiseuille assumption in equation 14.

### Reynolds Number and Wall Shear Stress (WSS)

The peak velocity in the aqueduct was noted, along with the peak Reynolds number, Wall Shear Stress (WSS) and pressure difference between isolated points in the lateral ventricle and fourth ventricle (see Figure 1 for location of measurement), Δ*P*. The peak Reynolds number, 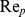, is based on the peak velocity 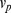 and hydraulic diameter 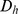. 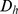 is given by 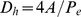, where *A* and 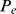 are the area and perimeter respectively. In this work, all cross sectional areas resembled an ellipse as 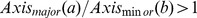 for all concerned cross sections. The perimeter of an ellipse was therefore used, and this is approximately given by 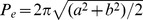. 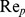 is therefore defined as 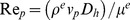, where 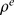 and 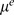 are the density and dynamic viscosity of CSF. Finally, the wall shear stress is defined by 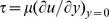. Here, 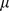 has already been defined and *u* is the flow velocity parallel to the wall and *y* is the distance to the wall.

### Application of Stenosis, ETV Outlet, EFV Outlet and FVOO Blockage

Owing to its powerful individual nodal manipulation capabilities, Blender was used to apply the local stenosis to the three-dimensional geometries for the cases involving the three degrees of HCP severity. The current standard voxel size produced from clinical imaging does not permit the accurate differentiation of different degrees of aqueductal stenosis. Finally, inlets and outlet boundaries were created using CFD-VisCART (ESI Group, Paris France. The final smoothed STL file for an open aqueduct is seen in Figure 2b.

As can be seen in Figure 1b, ETV is the location of perforation of the floor of the third ventricle during endoscopic third ventriculostomy. A boundary condition of 0 Pa was assigned.

There were three occlusion (atresia) possibilities taken into consideration. Firstly, the atresia of the bilateral foramina of Luschka was investigated, along with corresponding values for peak velocity in the aqueduct, Magendie and central canal. In addition to this, the WSS values at the surface of the aqueduct were investigated along with the previously defined pressure difference ΔP, in order to determine the extent of ventriculomegaly under FVOO.

Secondly the sole atresia of the foramen of Magendie was investigated along with corresponding values for peak velocity in the aqueduct, (average of the right and left) bilateral Luschka and central canal. Once again the WSS values at the surface of the aqueduct along with the pressure difference are recorded. Finally the occlusion of both of the aforementioned outlets was investigated along with same additional comparators previously outlined.

Atresia was simulated as a blocked outlet, whilst the application of EFV was to apply a concentric hole within the blocked outlet under consideration. As in the case for ETV, a boundary condition of 0 Pa was assigned.

### Solution Method and Post Processing

The governing multicompartmental poroelastic equations are solved with an implicit second-order central finite differences scheme on the midpoints and forward/backward Euler used on the boundary nodes. The quasi-steady time discretization (for the temporally dependent terms in the boundary conditions) is performed via a first-order Euler approach.

Flow through the multidimensional ventricles is solved using the multiphysics software CFD-ACE+ (ESI Group, Paris, France) which is based on the finite volume approach, along with central spatial differencing, algebraic multigrid scheme and the SIMPLEC pressure–velocity coupling. The coupling between the poroelastic solver and the flow solver is achieved through appropriate CFD-ACE+ user-defined subroutines.

Mesh generation (see Figure 2c) for the 3D volumes was achieved via the use of CFD-VisCART (ESI Group, Paris, France), which is an unstructured adaptive Cartesian grid generation system. CFD-VisCART offers a projected multiple domain method tree-based data structure to generate Cartesian-based non-conforming grids. The OmniTree data structure was used as it supports anisotropic grid adaptations [47]. The use of anisotropic solution adaptation is ideally suited for high gradient flow features and creates the potential for a dramatic reduction in the total number of cells to achieve a given level of solution accuracy.

The base discretisation for the spherically symmetric MPET model involved 81 nodes, as this yielded results within a 5% band from what we considered as the fully converged solution: full grid independence was achieved at 221 nodes. Similarly, adequate discretization of the 3D ventricular domain is required in order to acquire fully resolved results. Similar grid independence analysis resulted in meshes of over 2 million cells for that domain.

The coupling between the poroelastic solver and the flow solver is achieved through the use of CFD-ACE+s’ user-defined subroutines. After an initial assumption of the aqueductal flow, the pressures at the skull and inside the ventricles are passed to the user-defined subroutines as pressure boundary conditions (after each time step of the poroelastic solver). Following the Finite Volume solution of the ventricular and therefore aqueductal flow, the mass flux is calculated in the user-defined subroutines and passed to the poroelastic solver for the next iteration.

CFD-VIEW (ESI Group, Paris, France) is used as the post processing tool to analyze the flow physics (see Figure 2d and 2e). This tool also interacts with the CFD solver used in this work (CFD-ACE+).

## Results

### Aqueductal Stenosis

**Figure 4:**
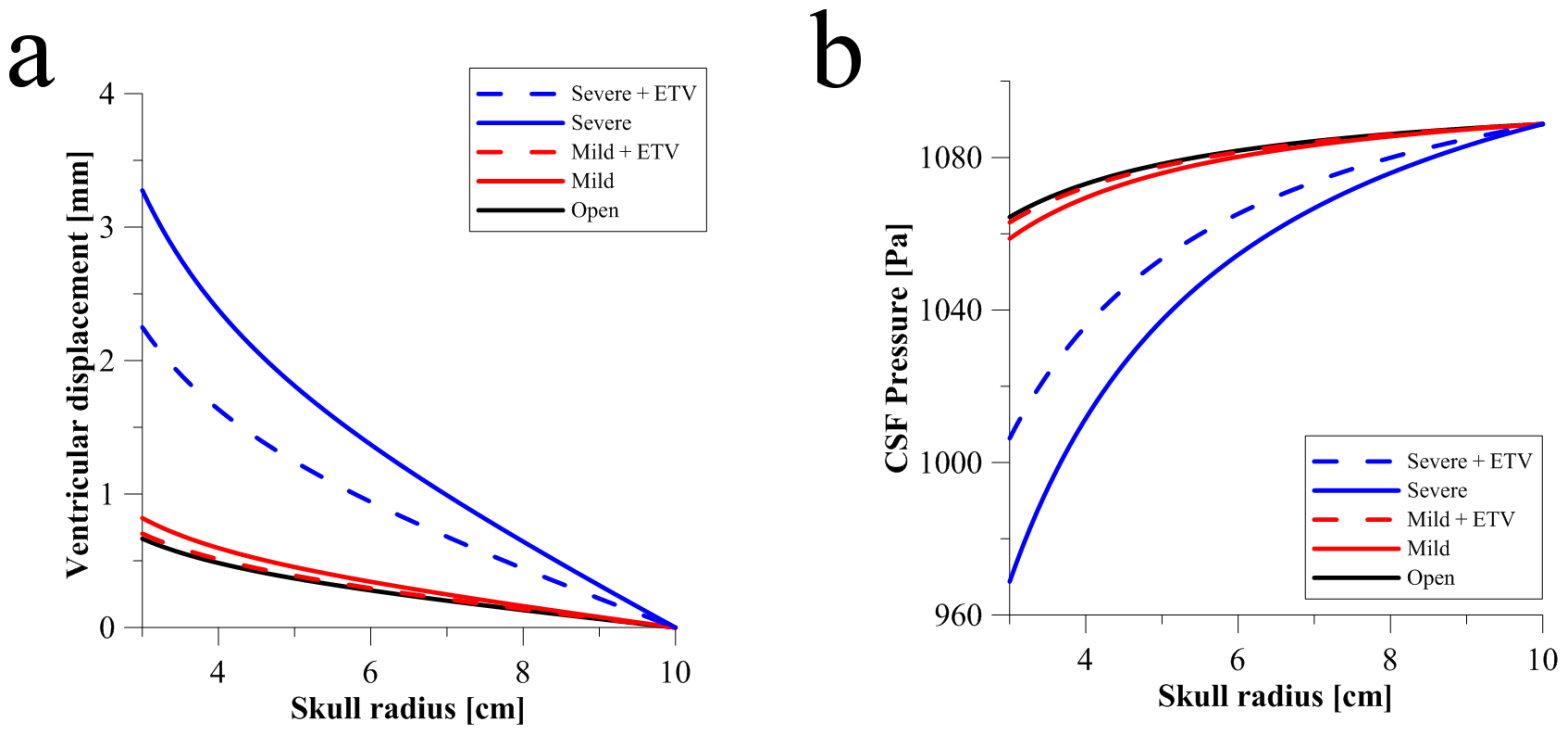
Comparing CSF pressure and ventricular displacement alleviation via ETV due to varying aqueductal stenosis. (a) Skull radius versus ventricular displacement for the open, mild and severely stenosed cases of the aqueduct. The application of ETV on the mild and severe case is also shown. (b) Skull radius versus CSF Pressure for the same cases of HCP severity and ETV application. Both plots were taken after *t = 50s*.

Figure 4 shows the application of the MPET model to obstructive HCP with the addition of ETV, along with the swelling factor of AQP4 being taken into account. The inclusion of the choroid plexus’ geometrical representation within the ventricular system and its coupling with the MPET model are also taken into account in these results. The hydraulic diameter, *d*, for the open, mild and severe cases is given in Table 1 as 3.00 mm, 1.25 mm and 0.80 mm respectively. It can be seen from Figure 4a that the ventricular displacement increases from approximately 0.67 mm in the open aqueduct to approximately 0.82 mm in the mild case of aqueductal stenosis. The severe case presents the highest level of ventricular displacement of around 3.28 mm.

The application of ETV substantially reduced the level of ventricular displacement to 0.70 mm for the mild case and around 2.25 mm for the severe case. This is a fair reduction in both cases, but especially for the mildly stenosed case, as its ventricular displacement level has been reduced to a level resembling the case of the open aqueduct. All of the aforementioned ventricular displacement values are taken at 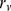. In addition, all of the displacements reduced to zero at the skull 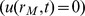 since the boundary condition imposed on the system at this point was that of a rigid, adult skull.

The CSF pressure variation to aqueductal stenosis shown in Figure 4b indicates a similar trend. There is a decrease from approximately 1064.38 Pa in the open case to, 1058.73 Pa in the mild case and finally reducing to 968.77 Pa in the severe case. The introduction of ETV increased the CSF Pressure from its decreasing levels to 1062.97 Pa and 1006.36 Pa for the mild and severe cases of aqueductal stenosis. It should also be noted that all CSF pressures converge to 1088.77 Pa on the skull, as expected, since this value corresponds to the absorption resistance boundary condition.

As can be seen from Table 2, the peak velocity in the aqueduct increases substantially between stenosis severity levels. In the open aqueduct, it is measured to be approximately 15.6 cm/s (Reynolds number of ∼ 524), whereas in the mild and severe cases, this increases to 45.4 cm/s (Reynolds number of ∼ 635) and 72.8 cm/s respectively (Reynolds number of ∼ 650). As expected, once ETV has been applied, the peak velocity in the aqueduct falls to 16.8 cm/s (Reynolds number of ∼ 235) and 17.1 m/s (Reynolds number of ∼ 153) for the mild and severe case respectively. The WSS values at the surface of the aqueduct display a similar trend. The peak WSS value at the surface of the open aqueduct was measured to be just less than 0.9 N/m^2^, whilst for the mild and severe cases of stenosis this increased to around 5.8 and 12.2 N/m^2^ respectively. The WSS was alleviated by the application of ETV (see Table 2), and this followed a decline from the aforementioned values to 1.5 N/m^2^ for both the mild and severe cases. The pressure difference between the lateral and fourth ventricles was used as a final comparator. As can be seen from Table 2, large fluctuations can exist with the application of aqueductal stenosis. The average pressure difference between the lateral and fourth ventricle was found to be roughly around 18 Pa. Once stenosis occurred, this increased from 18 to 94 Pa in the mild case and up to 241 Pa in the severe case. Once again the application of ETV helped rectify the large fluctuations back to normal levels, 15 and 20 Pa respectively. The velocity in the central canal did not exceed 2 cm/s for all above cases.

**Table 2:** The values of hydraulic diameter *D_h_*, peak velocity *v_p_*, peak Reynolds number *Re_p_*, wall shear stress (*WSS*) and pressure difference (between isolated points in the lateral ventricle and fourth ventricle) *ΔP,* for an open, mild and severely stenosed aqueduct of Sylvius.

Severity	*D_h_* [mm]	*v_p_* [cm/s]	*Re_p_*	*WSS* [N/m^2^]	Δ*P* [Pa]
Open	3.00	15.59	523.93	0.87	18
Mild	1.25	45.37	635.31	5.84	94
Mild+ETV	1.25	16.76	234.69	1.50	15
Severe	0.80	72.75	649.46	12.22	241
Severe+ETV	0.80	17.08	153.08	1.51	20

The above values once ETV has been applied are also given. All values obtained at *t = 50s*.

The porosity of the choroid plexus fluctuated between 0.675 and 0.775 for all cases in this study. The average velocity coming out of the entrance of the arteries to the choroid plexus was approximately 40 mm/s, given the boundary condition of 2 kPa at the inlet of the arteries.

**Figure 5:**
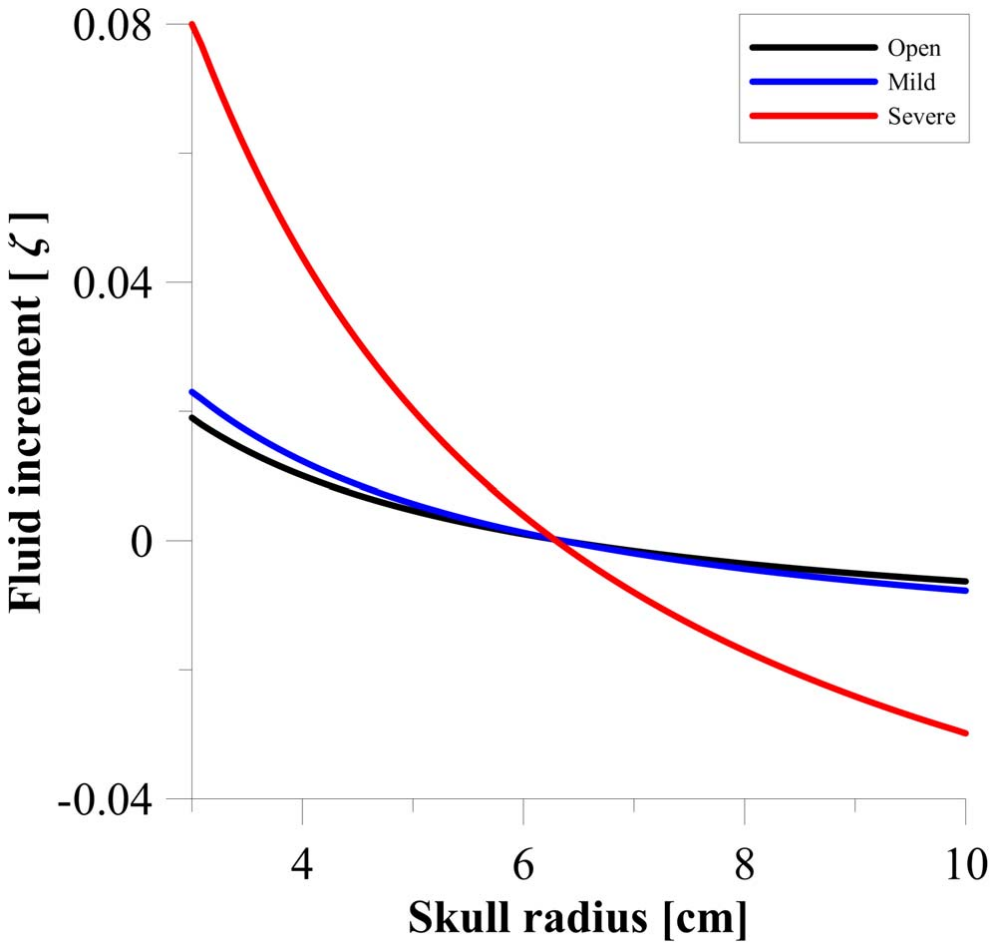
Depiction of the increment of CSF content (ζ). Increment of CSF content for different stages of aqueductal stenosis. Taken at t = 50s.

Figure 5 shows the change in CSF content (Equation 7) for the three cases of aqueductal stenosis. The primary aim of observing this relationship is to confirm that there is an expansion in the region close to the ventricles, and a subsequent compression against the rigid skull in the surrounding parenchyma. Negative values of ζ indicate fluid being squeezed out of the brain. The positive values of ζ indicates the existence of tissue damage adjacent to the cerebral ventricles. As expected, a larger degree of aqueductal stenosis corresponds to a larger CSF change in tissue adjacent to the ventricles and in addition, a large section of tissue (most likely grey matter) compression (between 7–10 cm). These results correlate well with other work in the literature [41], [44].

### Fourth Ventricular Outlet Obstruction (FVOO)

In addition to the implication of applied stenosis on the aqueduct, fourth ventricular outlet obstruction was investigated. This was done in order to help elucidate the consequence of applying EFV and comparing this to ETV.

**Figure 6:**
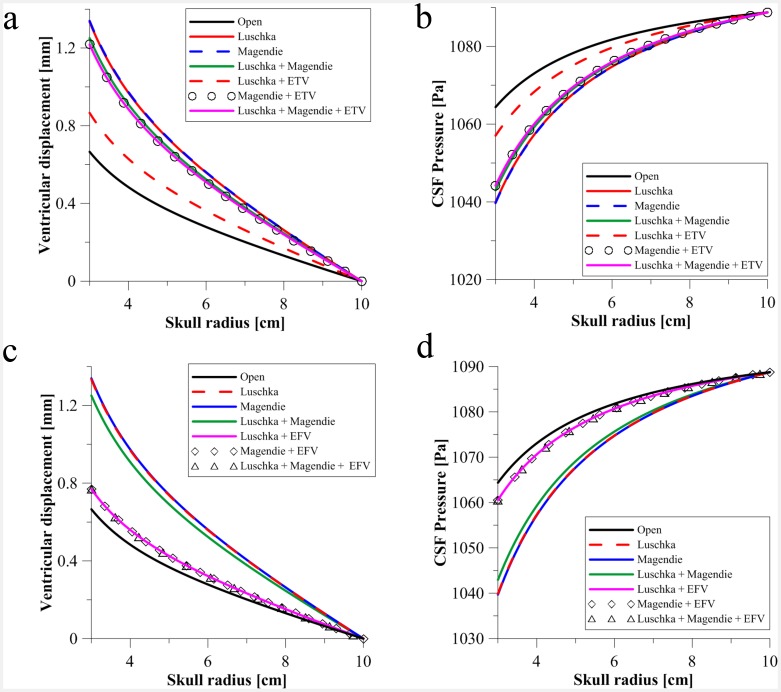
Comparing CSF pressure and ventricular displacement alleviation via ETV and EFV due to altering the site of occlusion on the fourth ventricle. (a) Skull radius versus ventricular displacement for induced occlusions in the foramina of Luschka and/or foramen of Magendie. The application of ETV on these cases is also shown. (b) Skull radius versus CSF Pressure for the same cases of selective occlusion with ETV application. (c) Skull radius versus ventricular displacement for induced occlusions in the foramina of Luschka and/or foramen of Magendie. The application of EFV on these cases is also shown. (d) Skull radius versus CSF Pressure for the same cases of selective occlusion with EFV application. Both sets were taken after *t = 50s*.

As can be seen from Table 3, occluding both the foramina of Luschka slightly increases the peak velocity from 15.6 cm/s in the open aqueduct to 16.5 cm/s. The peak velocity through the foramen of Magendie increases from 2.4 cm/s in the open case to 8.9 cm/s, followed in the same fashion by the peak velocity in the central canal, from 2.0 cm/s to 8.7 cm/s. The equivalent ventricular displacement for such an occlusion was 1.33 mm (see Figure 6), whilst the CSF pressure reduces from 1064.38 Pa to 1039.89 Pa. The pressure difference stays at ∼ 17 Pa, irrespective of this occlusion. This is in contrast to the stenosis of the Sylvian aqueduct which resulted in over a five-fold increase in the mild case alone. This contrast is further fortified by the similar WSS values at the surface of the aqueduct. The application of ETV and EFV to this dual occlusion has varying consequences. ETV approximately halves the peak velocity in the aqueduct whilst EFV keeps it approximately constant at 16.2 cm/s. As expected, since EFV is taking place in a closer vicinity to the occlusion, the peak flow rate through the foramen of Magendie (8.9 cm/s under the influence of this occlusion) shows a larger reduction than that of the ETV application (3.0 cm/s as opposed to 4.2 cm/s). The same trend applies to the central canal, where its initial peak velocity under occlusion of the bilateral foramina (8.7 cm/s) is reduced to 3.9 cm/s (ETV) and 2.6 cm/s (EFV) respectively. What is interesting is that ETV reduced the pressure difference between lateral and fourth ventricles quite considerably (from 17 Pa to 6.6 Pa), whilst EFV forces a slight decrease in this value (16.7 Pa).

**Table 3:** The values of peak velocity *v_p_* (in the aqueduct (*a*), Magendie (*m*), Luschka (*l*, average) and central canal (*cc*)), pressure difference between isolated points in the lateral ventricle and fourth ventricle, *ΔP* and wall shear stress (*WSS*) are given for the occluded foramina of Luschka and foramen of Magendie.

FVOO location	*v_p_(a)* [cm/s]	*v_p_(m)* [cm/s]	*v_p_(l)* [cm/s]	*v_p_(cc)* [cm/s]	Δ*P* [Pa]	*WSS* [N/m^2^]
Luschka (Lu)	16.52	8.90	Occluded	8.70	17	0.83
Magendie (Ma)	16.55	Occluded	2.93	2.62	17	0.82
Lu+Ma	15.01	Occluded	Occluded	35.48	16	0.63
Lu+ETV	8.92	4.22	Occluded	3.89	<10	0.29
Ma+ETV	9.04	Occluded	1.43	1.06	<10	0.33
Lu+Ma+ETV	8.47	Occluded	Occluded	5.59	<10	0.31
Lu+EFV	16.17	3.00	Occluded	2.62	17	0.79
Ma+EFV	16.17	Occluded	1.67	1.27	17	0.79
Lu+Ma+EFV	17.02	Occluded	Occluded	4.58	18	0.86

The peak velocity values for endoscopic third and fourth ventriculostomy (*ETV*, *EFV*) are also displayed. All values obtained at *t = 50s*.

The same applies to the WSS, since this is reduced from around 0.8 N/m^2^ to ∼ 0.3 N/m^2^, whilst in contrast it slightly decreases with the application of EFV (see Table 3). The application of ETV and EFV reduce the ventricular displacement to 0.87 mm and 0.77 mm, whilst the CSF pressure increased to 1057.04 Pa and 1060.57 Pa respectively.

The situation is similar for the occluded Magendie. The peak velocity through the aqueduct increases from 15.6 cm/s in the patent aqueduct case (Table 2) to 16.6 cm/s. The peak velocity through the bilateral foramina stays fairly constant at 2.9 cm/s, a slight increase from 2.4 cm/s in comparison with the same healthy case. The peak velocity through the central canal displays a slight increase from 2.0 cm/s to 2.6 cm/s, whilst the pressure difference remains relatively unchanged at ∼17 Pa. The ventricular displacement and CSF pressure remain identical to the case of the occluded bilateral foramina. The WSS value slightly decreases, from around 0.9 N/m^2^ to ∼0.82 N/m^2^. Comparing ETV and EFV for this occlusion, it is evident that ETV once again reduces the pressure difference with greater effect (from 17.4 Pa to 6.8 Pa), whilst EFV reduces it only marginally to 16.7 Pa. The peak velocities in the central canal, bilateral foramina and aqueduct are reduced to a greater extent with the application of ETV as opposed to EFV. Applying ETV and EFV reduced the ventricular displacement to just under 1.22 mm and 0.77 mm respectively. CSF Pressure increases to 1044.12 Pa for the ETV case, whilst for EFV it increases to 1060.56 Pa.

The final scenario involves the occlusion of all fourth ventricle exits except the central canal. The peak aqueduct velocity decreases slightly from 15.6 cm/s to 15.0 cm/s, whilst through the central canal it increases drastically from 2 cm/s to 35.5 cm/s. In addition, the WSS possesses a modest value of 0.63 N/m^2^. The pressure difference remains more or less constant, decreasing by just 2.1 Pa when compared to the open case. The ventricular displacement increases to approximately 1.25 mm, whilst the CSF pressure decreases to 1042.95 Pa.

**Figure 7:**
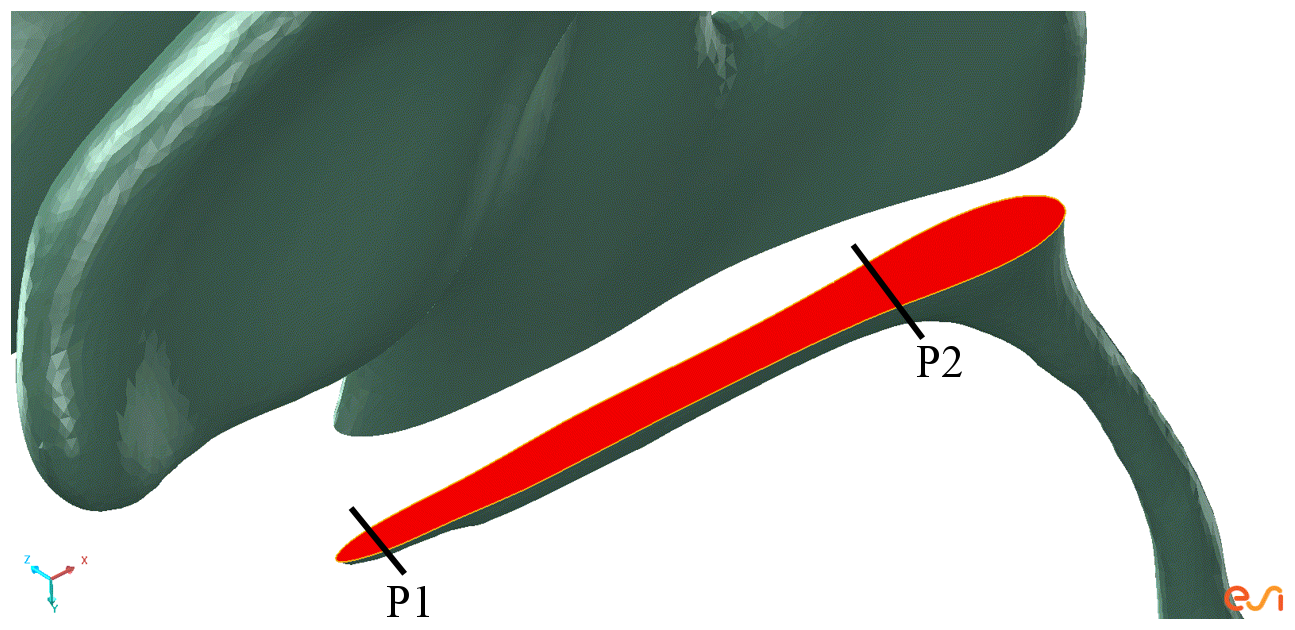
A segment of the lower region third ventricle used to obtain CSF velocity and pressure. The lower region of the third ventricle used to evaluate range of CSF velocity and pressure before and after ETV. The range was taken between points P1 and P2.

Applying ETV, we see a reduced peak velocity through the aqueduct to a greater extent than EFV (which increased compared to the combined obstruction of Luschka and Magendie); however EFV reduced the velocity in the central canal (4.6 cm/s as opposed to 5.6 cm/s). Finally, the pressure difference assumes a larger value due to ETV (∼6 Pa) as opposed to its competing neurosurgical technique (which raises it to ∼18 Pa). Further investigation in the vicinity of the ETV outlet was required to justify the lower than expected pressure difference between lateral and fourth ventricle. The velocity magnitude of CSF near the lower region (from just above the ETV outlet (point P1 on Figure 7) and linearly extending horizontally towards the aqueduct (point P2 on Figure 7)) of the third ventricle was noted to be in the range of 5.5 cm/s to 0.14 cm/s for the occluded Luschka’s (Occ. Lus.), 5.7 cm/s to 0.14 cm/s for the occluded Magendie (Occ. Mag.) and finally 5.4 cm/s to 0.13 cm/s for the tri-exit occlusion (Occ. Mag. And Lus.). Applying ETV, the respective ranges were: 8 cm/s to 0.065 cm/s (Occ. Lus.), 7.77 to 0.057 cm/s (Occ. Mag.) and 8.36 cm/s to 0.072 cm/s (Occ. Mag. And Lus.). The pressure range was also monitored in the same lower third ventricle region as that of the aforementioned CSF velocities. The pressures ranges before and after ETV were: from 21.0 Pa to 7.6 Pa (Occ. Lus.), 17.8 Pa to 6.9 Pa (Occ. Mag) and 80 Pa to 7.9 Pa (Occ. Mag. and Lus.). In the same order, the peak ETV outlet velocities were 12 cm/s, 11 cm/s and 12 cm/s respectively.

### Flow Visualization

**Figure 8:**
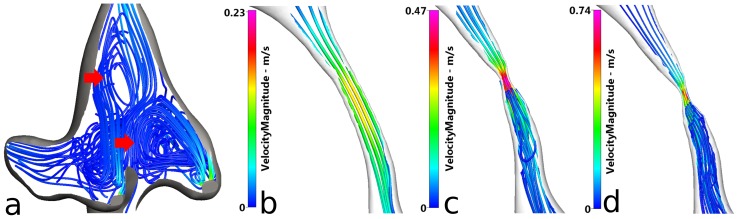
Velocity streamlines of the fourth ventricle and three cases of aqueductal stenosis (open, mild and severe). (a) View of the velocity streamlines of the aqueduct and fourth ventricle in a healthy patient in the sagittal plane. Two vortices are identified via the red arrows. (b) A more focused representation of the velocity streamlines of the aqueduct in a healthy patient. (c) Velocity streamlines of the mildly stenosed aqueduct. (d) Velocity streamlines of the severely stenosed aqueduct. CFD-VIEW was used to obtain all streamlines. All plots taken at *t = 15s*.

Figure 8a portrays the most complex flow found within the cerebroventricular system, namely in the fourth ventricle. As can be seen, two vortices (red arrows) have developed from the complicated partition of CSF flow arising from the aqueduct of Sylvius (which largely determines the nature of the CSF flow in the fourth ventricle), and they both rotate in an anti-clockwise direction. The other portion of CSF travels along the floor of the fourth ventricle and leaves via the three foramina (satisfying the second category of systolic centrifugal CSF flow). Flow exits through the foramen of Magendie and a comparatively low amount through the central canal. Figure 8b–d show representations of the velocity streamlines of an open, mild and severly stenosed aqueduct.

CSF flow was generally slow in the lateral ventricles and conformed to the centrifugal based classification outlined in Stadlbauer et al. [48]. In addition, areas of dead zones and recirculation were noticed in the lateral ventricles. The third ventricle obeyed the second category of systolic cranio-caudal flow, described in detail in the same literature.

## Discussion and Limitations

In this work, obstructive HCP was investigated via the gradual stenosis of the aqueduct of Sylvius (open, mild and severe), and the occlusion of individual or a combination of outlets of the fourth ventricle. The physical comparators used to describe the effects of this induced HCP include ventricular displacement, CSF pressure, peak velocities, pressure difference between lateral and fourth ventricle and wall shear stress. These comparators are also used to give an indication of the alleviating effects of the two surgical procedures proposed.

**Figure 9:**
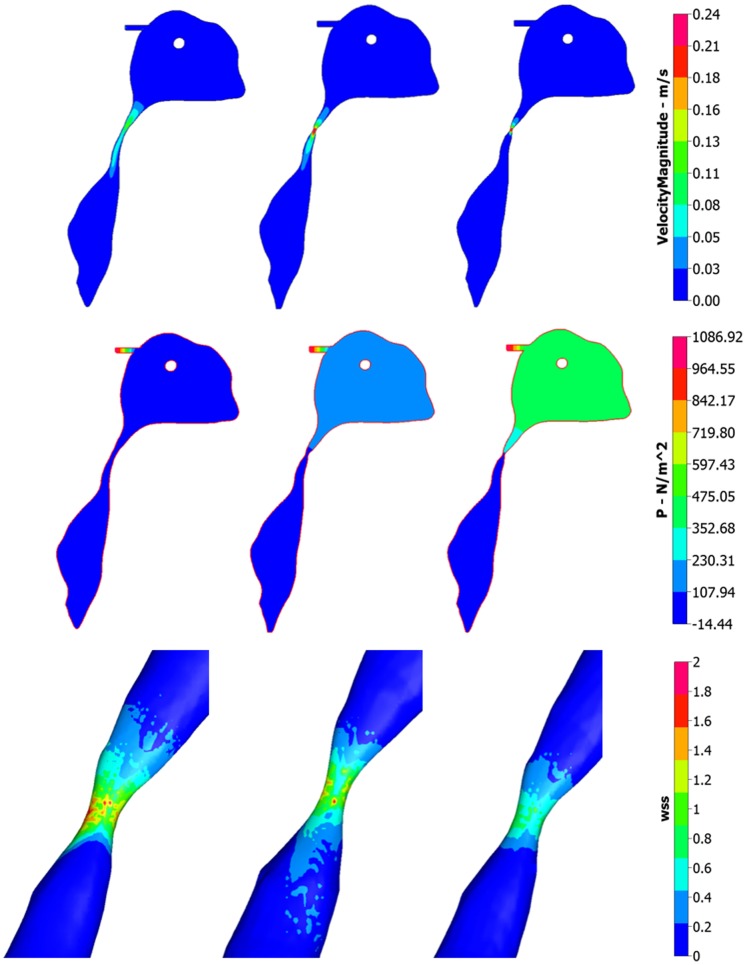
Comparing velocity magnitude, pressure (P) and Wall shear stress (WSS) for varying aqueductal stenosis. (First row) Sagittal view of the unobstructed, mild and severely stenosed aqueduct within the ventricular cavities. The contour lines denote velocity magnitude. Precise values for peak velocity within the aqueduct at the aforementioned stenosis levels can be found in Table 2. (Second Row) Pressure distribution of an extended ventricular region accompanying the open, mild and severely stenosed aqueducts. (Third row) WSS distribution of the mild, severe and mild with applied ETV cases of aqueductal stenosis. CFD-VIEW was used to obtain velocity magnitude, pressure and WSS. All plots taken at *t = 15s*.

It is evident from the results garnered in this study that the central canal does not simply act as a pathway for CSF flow. Gupta et al. [15] speculate that very little fluid passes through this ventricular boundary, and indeed this may seem to be the case under typical aqueductal stenosis (see Figure 9, first row). It was witnessed that the peak velocity through the central canal did not exceed 2 cm/s in all cases of aqueductal stenosis, and what was surprising was that this magnitude reduced with an accompanying increase in stenosis severity.

A noticeable difference is seen during the cases of FVOO, where the peak velocity in the central canal exceeded 35 cm/s during the combined occlusion of the bilateral foramina and the foramen of Magendie. The application of both ETV and EFV reduced the impact of the occlusions on the central canal for nearly all cases; however, ETV did so with greater success, as can be seen from the results in Table 3. The ventricular displacement was noted to increase with increasing severity of aqueductal stenosis, up to a maximum of 3.28 mm. It should be noted that since the main goal of this work was to compare the efficacy of both ETV and EFV interventions, the chronic levels of hydrocephalic development were not pursued here. This is however, within the scope and capability of the methodology [1]. In addition, the CSF pressure abided inversely to this notion, reducing to 968.77 Pa in the severe case.

Likewise, applying ETV at the floor of the third ventricle reduces both the ventricular displacement and CSF compartmental pressure to manageable levels. Figure 9 (third row) shows the effect aqueductal stenosis has on the WSS. The WSS values increase substantially from the open to the mild and severe cases. The peak velocity is raised 3 and over 4 times the value of the open case. Such observations are similar to the results of previous work done on the investigation of flow dynamics undergoing aqueductal stenosis [49], [50].

As the aqueduct tries to maintain the laminar CSF flow (350< Re <700) through the third and fourth ventricle by assisting in repelling the core of central fluid from the aqueductal wall effects [32], [49], a pressure gradient develops (see Figure 9, second row) to help maintain this flow of CSF (rising from around 18 Pa to 94 and 241 Pa in the mild and severely stenosed cases), and during aqueductal stenosis, gliosis could be perceived to induce the further narrowing of the stenosed perimeter [32], [51]. Such pressure gradients are also encountered in the literature [43], [52]. During atresia (by translucent membranes) of the bilateral foramina or foramen of Magendie, the pressure difference between the lateral ventricle cavity and the fourth ventricle remains at approximately 17 Pa. The effect that atresia of the fourth ventricular exits has on the CSF flow within the ventricular system is questionable. Provided that not all of the exits of the fourth ventricle are blocked (i.e. the central canal remains open), the peak aqueductal velocity would decrease by a relatively modest amount (15.0 cm/s), as in the case of the tri-exit closure (bilateral foramina and foramen of Magendie). The bulk flow increase would directly be associated with the central canal, as in the simulated case. The peak aqueductal velocity increased substantially to 35.5 cm/s.

One could appreciate that such a form of pronounced atresia could initiate secondary pathologies to FVOO, such as Syringomyelia. Indeed, recent developments indicate an increase in the expression of AQP4 in conjunction with the level of central canal occlusion and subsequent dilation [53]. This type of disorder could induce various forms of somatosensory impairment, as discussed by Hatem et al. [54]. Once the atresia is perforated during EFV, a largely reduced and constant aqueductal peak velocity is recorded. This is a moderate increase from that witnessed for the patent aqueduct. The central canal is alleviated from the burden of having to displace most of the CSF egress. Ventricular displacement is reduced to the same level for all cases of atresia during application of EFV, whilst the CSF pressure is elevated back to a similar level seen in the case of a healthy individual.

What was interesting is that although the management of the FVOO seems to be commensurate, it must be noted that ETV performed better in comparison, reducing: the average peak velocity in the aqueduct for the three cases of atresia, the pressure difference Δ*P* was reduced further, wall shear stress as well as ventricular displacement. CSF pressure was reduced below the levels expected from an individual with an open aqueduct. This fortifies the view of Mohanty et al. [38] that stipulate ETV should be the preferred treatment for FVOO. EFV can be deemed as a viable alternative, especially when cases such as the one described by Gianetti [37], where the foramen of Monro and third ventricle did not satisfy prerequisite size constraints for suitable ETV adaptation. It must be noted that the basis of the accuracy of the CSF flow within all four ventricles is entirely dependent on the accuracy of the MRI acquisition process. The anatomic MRI scans cannot help elucidate the fine surface structures present on the choroid plexuses, as these characteristics are of a scale of µm. The choroid plexus representation is seen in Figure 1b.

A simplified boundary condition of 2000 Pa for the arteries was utilised for conservation of computational resources. Unlike previous work, the amount of CSF produced in the lateral, third and fourth ventricles was assumed identical, and not confined to a ratio in terms of total CSF produced. However, there is general agreement that there is no literature outlining the exact proportions of CSF production in the various choroid plexus sites [15].

The unique permeability of the CSF compartment (*k_e_*) was relaxed in this study, and given way to a fluctuating adaptation represented by equation 6. The reference pressure was arbitrarily chosen to have a value of 1 kPa. If CSF pressure *P_e_ <P_ref_*, then the permeability increases, whilst the converse is true for *P_e_ >P_ref_*. Strictly speaking, AQP9 is also found in astrocytes and may possess a synergy in aiding AQP4 [55], however, not much else is known about this aquaglyceroprotein to warrant further consideration in this type of setup. The aim of this varying permeability compartment was to bring to light a very simple feedback mechanism that would theoretically counteract the effects of ventricular dilation and subsequent elevations of CSF pressure through the efflux of excess CSF to the blood system [56].

The visualisations of the cranio-caudal flow are in good agreement with the categorised CSF flow patterns found using time-resolved 3D PC-MRI velocity mapping utilising the capture of pathlines from 3D PC-MRI data (there was no inclusion of driving pressure fields). It should be noted that the aforementioned velocity mappings were not used on any volunteers suffering from hydrocephalic symptoms, and it was stressed that the effect of aging on the CSF flow categorisations could be ignored [48], [57]. Stadlbauer et al. [48], [58] has outlined the detailed method of ascertaining the truly complicated nature of CSF flow in a variety of normal and hydrocephalic patients through the extended use of 3D PC-MRI. The segregation of the complicated nature of CSF flow has been attempted here, and their classifications warrant consideration of the ventricular geometry and its finer features, such as the interthalamic adhesion. In order to make inferences regarding the efficacy of EFV and ETV would require a large sample size to ensure various geometrical variations are catered for.

One of the main reasons for considering detailed CSF flow categorisations is that areas of interest such as CSF flow diversion techniques (ETV, EFV, shunting, intracerebroventricular drug administration etc.) for treating obstructive HCP can only benefit from more detailed qualitative (and quantitative where applicable) CSF flow observations and categorisations. For instance, CSF flow dynamics in known to be influenced by the position of the interthalamic adhesion (see Figure 1), and it is postulated that it may effect the development of HCP, since the relative position of the adhesion to the aqueduct induced higher pressure gradients in the third ventricle [59].

Levine [43] suggests that a “mini-gradient” of around 133 Pa, combined with irregular tissue compliance may lead to HCP. A similar statement is also expressed by Penn & Linninger [60], where they indicate that only small pressure differences are needed to instigate ventricular enlargement. The conclusions from the aforementioned authors support what we have obtained from conducting our simulations. For aqueductal stenosis, we can see from Table 2 in our manuscript that the change in pressure between lateral and fourth ventricle increases with degree of stenosis, however once ETV takes place, Δ*P* drops back to levels similar to pre-stenosis states. The change in pressure is heavily influenced by very high degrees of stenosis, as expected from the governing MPET formulation. During FVOO however, we see a seemingly constant pressure difference of around 20 Pa present. Treating individual cases of obstructed Luschka or Magendie with ETV reduces Δ*P* to levels below those witnessed by an unobstructed configuration. This could prove to induce adverse therapeutic effects for alleviating the symptoms of HCP, and would prove an interesting scenario to validate experimentally.

The pressure difference between lateral and fourth ventricle indicates an area that requires further investigation. This is especially important for FVOO cases treated with ETV. Our results showed lower than expected levels of WSS and peak aqueductal velocity. Once applied, ETV also manages to increase the CSF velocity in the vicinity of the outlet by an average of 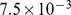 cm/s in all cases involving FVOO. In the vicinity of the aqueduct, the CSF velocity is reduced by an average of 0.07 cm/s. This phenomenon is due to the ETV outlet itself having a high CSF exit velocity (∼12 cm/s). The simplified ETV outlet boundary condition (0 Pa) will therefore need to be investigated in detail in future work, as its effects are evident. This future work should also include a simultaneous CFD analysis (directly applying boundary conditions from 3D-PC MRI results) of CSF production rates in close proximity to the CP’s of the ventricles. We also predict that there is strong anatomic correlation to the ETV orientated results, and so the effects of the interthalamic adhesion should be investigated since the velocity is low near the aqueduct when considering this lower portion of the third ventricle. However, aqueductal velocities are much higher in the aqueduct itself, so the bulk of the flow must be diverted from the geometrical aspects of the third ventricle.

The limitations surrounding the proposed MPET model include the use of a linear stress-strain relationship in light of evidently large strains. A spherically symmetric geometry which could theoretically prohibit the interpretation of the order of dilation amongst the various ventricular cavities is also an area of improvement. The simplification to quasi-stationary equations assumes altered pulsatility in the environment and does not play a role in hindering the investigation of properties over the time scale of HCP. In order to more accurately investigate their role, the more general transient equations must be solved. A constant isotropic permeability is also assumed, barring the possible effects of investigations of the diminishing of cerebral blood flow. The use of constant material parameters, such as elastic properties of the parenchyma, compartmental porosity, permeability and transfer coefficients is an additional limitation.

The quadruple MPET model introduces an additional ten parameters that are unknown; these are the Biot parameters and permeabilities of the additional blood networks, and the transfer coefficients between the fluid networks. As far as the authors are aware, no suitable experimental data exist and approximations are made through an extensive parametric search detailed in Tully & Ventikos [1], as outlined in the *Methods* section. Current MRI techniques utilizing single point ramped imaging with T1 enhancement have been used to assess the porosity of sandstone [61]. The problem here is that the spatial resolution is of the order of millimetre and sub millimetre levels, and has not been attempted on living tissue. The permeability, 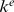 and Biot parameter, 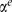 of the interstitial fluid network are approximated in the literature and we adopt the same values as published in [44]. However, such data are rare for parenchyma tissue and it is well known there are intrinsic difficulties in applying traditional mechanical tests to tissue. This is particularly true when the tissue is likely to have ‘active’ transport mechanisms that will not be functional in excised tissue. Clinical studies show that there is an observable difference in the properties of the white and grey matter [62], [63]. However, there is a lack of appropriate experimental data to quantify these differences, hence, many current models treat the parenchyma as a homogeneous tissue [1], [46], [52], [64]–[67].

Promising studies using magnetic resonance elastography (MRE) calculate the shear stiffness of cerebral tissue *in vivo*; however, they are yet to establish consistent and agreeable values. Evidence of this is that the reported numbers differ by orders of magnitude [63], [68], [69]. Sack et al. [68] assume a homogeneous tissue, and do not account for the inherent differences in white and grey matter. Green et al. [70] on the other hand, account for these differences in their calculation of load and storage modulus. We agree with others in the field [71] that MRE is dependent on the assumptions already made to the tissue properties, which in turn promote inconsistencies. In addition, the lack of evidence surrounding the acquisition of shear stiffness values via the extrapolation from higher to lower excitation frequencies is also an area worthy of further investigation.

The inclusion of the choroid plexuses in the region of the lateral, third and fourth ventricles, along with a pressure inlet boundary condition of 2 kPa (15 mmHg) at these locations substantially increased the magnitude of the peak CSF velocity flowing through ventricular cavities (with the most pronounced effect being in the aqueduct). This should be expected as this is the first time that this level of detail is attached to the inlet boundary conditions of the regionally accurate choroid plexuses, and in addition, at all CSF producing sites (lateral, third and fourth ventricles) of the ventricular system. The level of porosity and permeability at the surface of the choroid plexuses plays an important role in ultimately determining the egressing CSF velocities; however, there is an evident lack of experimental data surrounding these values. The value of 2 kPa for the aforementioned boundary conditions arises from generally accepted pressure values in the relevant conflated feeding arteries. Work conducted by Bammer et al. [72] and Wetzel et al. [73] allowed for the intracranial arterial hydrodynamics to be elucidated upon through the use of time-resolved 3D PC-MRI and flow sensitized 4D MR. Peak systolic flows of around 71 cm/s were found in different vascular segments [73]. For the PCA for instance, which feeds the lateral ventricles, velocities as high as 63 cm/s were outlined [72]. Being able to accurately depict the driving mechanisms of CSF production in the form of the choroid plexuses is therefore a difficult task, as this would require the use of pressure fields and therefore pressure boundary conditions. The use of the 2 kPa boundary condition is, in our opinion, a fair and accurate starting point to deliver a qualitative understanding of the interplay regarding CSF production. Currently, reliable non-invasive *in vivo* pressure measurements are not commonly obtained with current technological means, and so this virtual boundary condition was chosen for simplicity, since the subarachnoid space was not taken into consideration.

There is currently no clinical literature that allows for a comparison of the MPET model, with all the sites of CSF production present to be compared. It would also be taxing to suggest at this point in time, that a comparison of the ETV and EFV procedures on artificially induced aqueductal stenosis and FVOO be made with clinical data. To the authors’ knowledge, no MRI obtained velocities exist in these circumstances for such a comparison to be made. Shunting on the other hand, would have proven easier to compare to, however, there is an exhaustive range of literature using shunting as opposed to ETV as a method of alleviating the symptoms of aqueductal stenosis, and none for EFV alleviating the symptoms of FVOO.

## Conclusions

This study presents the first assessment of the impact of aqueductal stenosis and fourth ventricle outlet obstruction along with the applications of ETV and EFV, on an anatomically accurate representation of the cerebroventricular system. A simple relationship relaxing the constraint of a unique permeability for the CSF compartment in order to account for the Aquaporin-4 swelling characteristics has also been incorporated into this MPET framework which can allow for the intrinsic investigation of multiscalar, spatio-temporal transport of fluid between cerebral blood, CSF and the brain parenchyma.

The findings in this study support the appeal of ETV in cases of mild and severe aqueductal stenosis, as the metrics of comparison used in this work show an appreciable reversal of the effects of stenosis. FVOO is a new case taken under consideration, and the comparison of ETV and EFV on the different exit sites of the fourth ventricle proves to be more intricate. The results from this study outline that the greatest reversal of the effects of all three forms of atresia come by opting for EFV rather than ETV. However, our ETV findings indicate levels of aqueductal velocity and pressure difference between lateral and fourth ventricles to be low in comparison to the normal case. A full understanding of these effects will require a detailed estimate of ETV outlet boundary conditions, with the use of 3D PC-MRI in order to assess the complex CSF dynamics in the region. The interthalamic adhesion has the potential to alter the CSF dynamics in the third ventricle, and so this investigation should preferably be done simultaneously.

Other methods that could influence the CSF flow such as shunt placement, can be easily incorporated into this current framework. The addition of the aquaporin swelling characteristics into this detailed model allows the initiation of a formal discussion on how these channels influence CSF transport. Pharmaceutical regulation of AQP’s may also be incorporated into such an extendable mathematical framework.
